# Examining relationships between delay and probability discounting and perceived stress in a high-income online sample

**DOI:** 10.1007/s10865-026-00711-0

**Published:** 2026-09-03

**Authors:** Mary J. King, Michelle Rockwell, John Epling, Jeffrey S. Stein

**Affiliations:** 1 Graduate Program in Translational Biology, Medicine and Health, Virginia Tech, Blacksburg, VA USA; 2 Fralin Biomedical Research Institute at Virginia Tech Carilion, 2 Riverside Circle, Roanoke, VA 24016 USA; 3 Department of Family & Community Medicine, Carilion Clinic and Virginia Tech Carilion School of Medicine, Roanoke, VA USA

**Keywords:** Delay discounting, Probability discounting, Stress, Survey

## Abstract

Previous studies have shown a positive association between perceived stress and delay discounting (DD; devaluation of a consequence as a function of its delay); individuals experiencing high levels of stress tend to prefer smaller, immediate rewards over larger, delayed rewards. However, the link between perceived stress and DD may be confounded by their mutual and well-documented association with socioeconomic disadvantage (e.g., low income, scarcity). Moreover, few studies have examined the association between perceived stress and probability discounting (PD; devaluation of probabilistic outcomes), which is also reliably associated with socioeconomic disadvantage. To isolate the association between perceived stress, DD, and PD from socioeconomic disadvantage, the current study recruited a sample of US adults (N = 247) reporting minimal income constraints (i.e., household incomes ≥ $100,000/year) on Prolific, an online crowdsourcing platform. Participants completed DD and PD assessments for two separate reward magnitudes ($100 and $10,000), the Perceived Stress Scale (PSS), and a demographic questionnaire. In multiple regression, greater perceived stress was associated with higher DD rates for $100 (*p* < .05) and PD rates for $100 (*p* < .01); however, perceived stress was not significantly associated with DD or PD for $10,000. Thus, the association between perceived stress and both DD and PD appears independent of socioeconomic disadvantage, although our findings suggest that these associations are restricted to smaller reward magnitudes. Future research is needed to examine the association between these factors across varying populations, and how elevated stress levels may negatively impact real-world decision-making involving delay and uncertainty.

**Supplementary Information:**

The online version contains supplementary material available at https://doi.org/10.1007/s10865-026-00711-0.

## Introduction

Delay discounting (DD) is associated with a wide range of maladaptive health behaviors and clinical disorders, including tobacco and other substance use (Bickel et al., 1999), nonadherence to preventive and treatment services (Epstein et al., 2021; Vaughn et al., 2021), as well as psychiatric conditions such as major depressive and bipolar disorders (see Amlung et al., 2019 for meta-analysis). DD describes the devaluation of a consequence as a function of its delay. For example, individuals with high DD levels often prefer smaller, immediate rewards (e.g., $50 now) over larger, delayed rewards (e.g., $100 in 6 months). Probability discounting (PD) is a similar concept but describes the devaluation of a consequence as a function of its risk: for example, when given a choice between receiving $50 with 100% certainty or $100 with 60% certainty, an individual with high PD levels would likely prefer the guaranteed, although lesser, reward. In prior research, PD is associated with many of the same maladaptive health behaviors evident in the study of DD (e.g., nicotine use, Nolder & Anderson, 2023), but considerably less research has investigated this decision-making process compared to DD and extant findings appear less robust.

Perceived stress is also a relevant factor in decision-making, reflecting the degree to which individuals perceive events in their life as uncontrollable, unpredictable, or overwhelming over a given period (Cohen et al., 1983). Unlike physiological or other subjective measures of stress, perceived stress reflects the combined impact multiple stressors—both acute and chronic. In previous observational studies, greater perceived stress has been cross-sectionally associated with higher DD in adults and adolescents in the general population, as well individuals in recovery from substance use disorders (Fields et al., 2009, 2014; Malesza, 2019; Tomlinson et al., 2020). In contrast, experimental studies have not observed reliable effects of acute stressors on DD compared to non-stress or placebo controls (Forbes et al., 2024). Although seemingly inconsistent with the observational findings on perceived stress, we note that experimental research is limited to investigating the acute impact of relatively mild stressors (e.g., public speaking, cold exposure), which may not capture the cumulative burden of stress and broader subjective experiences reflected in perceived stress. In addition, in contrast with the larger literature on DD and stress, far less research has examined associations between DD and measures of stress, either observationally or experimentally (e.g., Lempert et al., 2012). A few studies have examined the broader construct of risk aversion, but these studies have primarily used experimental designs and are again limited by examining the effects of acute exposure to a single, mild stressor. For example, Sokol-Hessner et al. (2016) found no significant effect of acute stress on risk attitudes in a simple gambling task, but Buckert et al. (2014) found that participants undergoing a stress induction protocol were more likely to take risks for economic gains. Thus, further investigation is needed to understand how PD and perceived stress may be related.

The extent to which the associations between DD and perceived stress are confounded by income-related covariates is unclear. Both perceived stress and DD are reliably negatively associated with income. For example, people with lower income or who are experiencing poverty show higher DD rates (Wan et al., 2024), and individuals concerned with things like financial stability and food affordability are more likely to experience high perceived stress levels (Parthasarathy et al., 2022). Likewise, experiencing negative income shock (i.e. abrupt loss of income) is associated with higher DD rates in observational studies (Downey et al., 2023). These findings are also evident in experimental research, where simulations of negative income shock using laboratory-based economic tasks or brief vignettes increase both DD and perceived stress (Bickel et al., 2016; Haushofer & Fehr, 2014).

One way to control for the potential confounding effects of a “third variable”, such as income, in observational research is to recruit a study sample with limited variability on that dimension (Rothman et al., 2008). Here, restriction of that variable serves to minimize its potential confounding influence in observed associations. Accordingly, our objective in the present study was to examine whether perceived stress is associated with DD and PD in participants with household incomes of at least $100,000, which restricts the investigation to approximately the highest two income quintiles in the United States (Kollar & Scherer, 2025). This minimizes the potential influence of more extreme socioeconomic disadvantage. Because substantially more research has examined DD compared to PD in the context of perceived stress (as reviewed above), primary analyses focused on DD and perceived stress, while exploratory analyses examined PD and perceived stress. Discounting of two separate reward magnitudes ($100 and $10,000) were examined to evaluate the robustness of associations between perceived stress and discounting rates, as prior research shows that both DD and PD vary with reward amount (e.g., Cox et al., 2020; Green et al., 2013). In a single survey session, we assessed perceived stress, as well as DD and PD at two reward magnitudes ($100 and $10,000). We hypothesized that perceived stress would be positively associated with DD rates at either magnitude, where those reporting higher perceived stress would be more likely to choose the smaller, immediate reward (more delay averse), and that PD rates at either magnitude would be negatively associated with perceived stress, where those with higher perceived stress would be less likely to choose the smaller, certain rewards (more risk prone).

## Methods

This study was conducted as part of a broader line of research by our group comparing decision-making in primary care healthcare providers to non-healthcare providers (e.g., Epling et al., 2026). The data reported here are those from a cohort of non-healthcare providers, recruited online. To facilitate direct comparisons to primary care clinicians, the present study was restricted to adults with a) household incomes of $100,000 in order to align with the higher income levels typical of primary care physicians, and b) employment outside of medicine or closely-related fields.

### Participants

Participants were recruited using Prolific (www.prolific.com). The survey was only available to individuals whose Prolific user profile indicated the following: a) employment sector was in any category other than “Medicine” or “Science, Technology, Engineering, & Math”, b) between the ages of 28 and 75, c) combined household income of at least $100,000, and d) located in the US. There were no other restrictions applied to the Prolific sample outside of these demographic characteristics. Participants provided electronic informed consent as approved by the overseeing Institutional Review Board.

### Procedure

Participants completed the online survey through Qualtrics Survey Software (Qualtrics, Provo, UT). As detailed below, the survey contained discounting tasks, a perceived stress assessment, and demographic questions. Participants were informed at the beginning of the survey that it would take approximately five minutes to complete. The order that participants were shown the discounting tasks was randomized in order to account for potential order effects. DD and PD were assessed for two reward magnitudes of the delayed or probabilistic reward ($100 and $10,000) due to well-established findings in which DD and PD rates vary by reward magnitude. That is, in the assessment of DD, larger rewards are discounted at lower rates compared to smaller rewards (Cox et al., 2020; Green et al., 2013); in contrast, in the assessment of PD, this effect is reversed, so that larger rewards are discounted at higher rates (Cox et al., 2020). Assessing DD and PD rates at both magnitudes allows internal validation of the present study’s assessments by providing the opportunity to replicate these “magnitude effects” while also allowing for the examination of differential sensitivity of different reward magnitudes (e.g., due to floor or ceiling effects in DD or PD rates). Assessment of two different reward magnitudes allows for both 1) evaluation of the association between discounting and perceived stress under varying economic rewards and 2) a validity check, as prior studies have reliably shown magnitude effects for both types of discounting assessed (Cox et al., 2020; Green et al., 2013).

#### DD tasks

The 6-trial adjusting-delay discounting task (Koffarnus et al., 2021; Stein et al., 2022) was used to assess DD by arranging hypothetical choices between smaller, immediate and larger, delayed monetary rewards. This task has been adapted from its original 5-trial version (Koffarnus & Bickel, 2014) to increase the resolution and range of measurement of DD. Participants completed two separate iterations of the task to examine discounting of the two reward magnitudes, which were shown in a randomized order. The first trial of each began with a choice between the larger amount ($100 or $10,000, depending on task) after a delay or half of the amount ($50 or $5,000, depending on task) immediately. After the first choice had been made, the delay shown in subsequent trials would decrease if the smaller, immediate option was chosen or increase if the larger, delayed option was chosen (Downey et al., 2025). Participants were instructed prior to starting the trial that these rewards were hypothetical, but that they should make their choices as if the rewards were certain.

The larger reward’s delay was adjusted up or down across trials (increased after choosing the larger option, decreased after choosing the smaller option), with the final adjusted delay expected to produce indifference between options. The adjusted delay value following the final trial, which could range from approximately 26 s to 52 years, was extracted as a measure of the effective delay 50 (ED50), which is the delay required to reduce the subjective value of a given reward by 50% (Yoon & Higgins, 2008). Higher ED50 values indicate lower rates of DD, meaning there is a greater valuation of the delayed outcome. The inverse of the ED50 value serves as the DD rate parameter (*k*), which describes the steepness of the DD curve. Higher values of *k* indicate higher rates of DD, or greater devaluation of delayed rewards (Koffarnus et al., 2021). All *k* values were natural log-transformed prior to analysis in order to reduce positive skew, so that ln *k* served as our final DD rate parameter.

#### PD tasks

The 6-trial adjusting-probability discounting task (Downey et al., 2023; Dwyer et al., 2023; Koffarnus et al., 2021) was used to assess PD. These procedures were identical to that of the DD task, with the following exceptions. All rewards were immediate, and the probability of receiving the larger reward was adjusted across trials. The first trial of each PD task iteration began with a choice between having a 50% chance of receiving a larger amount ($100 or $10,000, depending on task) or a 100% chance of receiving the smaller amount ($50 or $5,000, depending on task). Participants were instructed prior to the first trial that these choices should be perceived as immediate.

The larger reward’s probability was adjusted up or down across trials (increased after choosing the smaller option, decreased after choosing the larger option), with the final adjusted probability expected to produce indifference between options. The size of the adjustment started at 25% after the first trial and was reduced by half after each subsequent trial, with probabilities rounded to the nearest whole percentage value. The adjusted probabilities after the final trial, which could range from approximately 1% to 99%, were converted to odds against receiving the reward (odds against = [1-*p]*/*p*]). The inverse of this odds against value served as the PD rate parameter (*h*), which describes the steepness of the PD curve (Koffarnus et al., 2021). Higher values of h reflect greater devaluation of probabilistic rewards. All *h* values were natural log-transformed prior to analysis in order to reduce positive skew, so that ln *h* served as our final PD rate parameter.

#### Quality control

An attention check was included at the end of each of the four discounting tasks. For both DD tasks, participants were asked whether they would prefer to receive the smaller amount now or the larger amount now, a method used in previous studies of discounting (Craft et al., 2022; Freitas-Lemos et al., 2023; Stein et al., 2022). Similarly, for both PD tasks, participants were asked if they would rather have a 100% chance of the smaller amount or a 100% chance of the larger amount, again with the assumption that the larger would always be preferred. Choices for the smaller reward in either attention check were interpreted as inattention or valuation of money that was incompatible with our method of measuring DD and PD.

#### Perceived stress scale

The 10-item Perceived Stress Scale (PSS), a common measure of personal stress, was used to estimate individual stress levels (Cohen et al., 1994). Items containing questions of how the participant has felt related to the stress they may have experienced over the past month (e.g., “In the last month, how often have you felt nervous and stressed?”) were rated on a 5-point scale, ranging from “never” (0) to “very often” (4), so that higher scores were associated with higher perceived stress. Four of the items were reverse scored (e.g., “In the last month, how often have you felt that things were going your way?”). Ratings on each of the items were summed so that higher scores indicated higher perceived stress, and scores could range from 0 to 40. Internal consistency reliability for the 10-item PSS was assessed using Cronbach’s alpha for the overall scale.

#### Demographic questions

The demographic questionnaire included age, education level, gender, and combined household income (CHI). Age and CHI were both used as part of the pre-screening done through Prolific, but were verified within the survey and to be included in analysis.

### Statistical analysis

Participant characteristics are summarized using medians (IQRs) for age, and frequencies (percentages) for all other characteristics. PSS scores were summarized based on their category (low, moderate, or high perceived stress), but the raw scores were used for analysis. R version 4.2.1 was used to conduct all statistical analyses. Specifically, the *tidyverse* package was used for data cleaning, and the *gtsummary* and *ggpubr* packages were used for data visualization.

Paired *t* tests were used to compare DD and PD rates (ln *k* and ln *h*, respectively) between the two reward magnitudes. Multiple linear regression was used to examine associations between PSS scores (explanatory variable) and both ln *k* and ln *h* (outcome variables) in separate regressions. Age, education, gender, and annual combined household income as additional explanatory variables, as these were deemed relevant demographic variables to examine based on their previous association with discounting (Downey et al., 2025; Göllner et al., 2018; Reimers et al., 2009). We also ran univariate analyses for each of these. Participants with DD (ln *k*) or PD (ln *h*) values that were deemed outliers (defined as values >3 standard deviations from the mean) were excluded from the primary regression analysis for that reward magnitude. Sensitivity analyses were conducted by repeating each regression with all outliers included.

## Results

### Participant characteristics

Participants completed the survey during August 2024, with a median completion time of 5.89 min (IQR 4.41 – 7.57 min). A total of 247 participants completed the survey, but ten participants (4.05%) were excluded from all analyses for having one or more failed attention checks across the four discounting tasks. Additionally, fifteen participants (6.07%) were excluded from all analyses for self-reporting an income below $100,000, which was inconsistent with the income that had been previously reported to Prolific. The remaining 222 participants (89.88%) were eligible to be included in the analysis. For each of the four regression analyses, outliers were identified as those with discounting values more than three standard deviations away from the mean, and were excluded from that individual analysis.

Demographic characteristics and PSS scores are shown in Table 1. The median age of participants was 48 years old (IQR 39, 58). The majority of the sample identified as female (121/222, 54.50%) and self-reported an annual combined household income (CHI) between $100,000 and $149,999 (124/222, 55.86%), based on the income values that were self-reported in our survey. The median PSS score was 14 (IQR 8, 20), which would indicate “moderate” perceived stress. Reliability analysis using Cronbach’s alpha showed “excellent” internal consistency for the overall PSS (α = .93).

**Table 1:** Participant characteristics

Characteristic	N = 222^1^
Age	48 (39, 58)
Education	
2-year degree or less	45 (20.27%)
4-year degree	98 (44.14%)
Grad/professional degree	79 (35.59%)
Gender	
Male	101 (45.50%)
Female	121 (54.50%)
CHI ($1000)	
100–149.9	124 (55.86%)
150–299.9	88 (39.64%)
300–449.9	7 (3.15%)
450+	3 (1.35%)
PSS score	14 (8, 20)

^1^Median (Q1, Q3); n (%)

### Regression analyses

#### Delay discounting

**Fig. 1:**
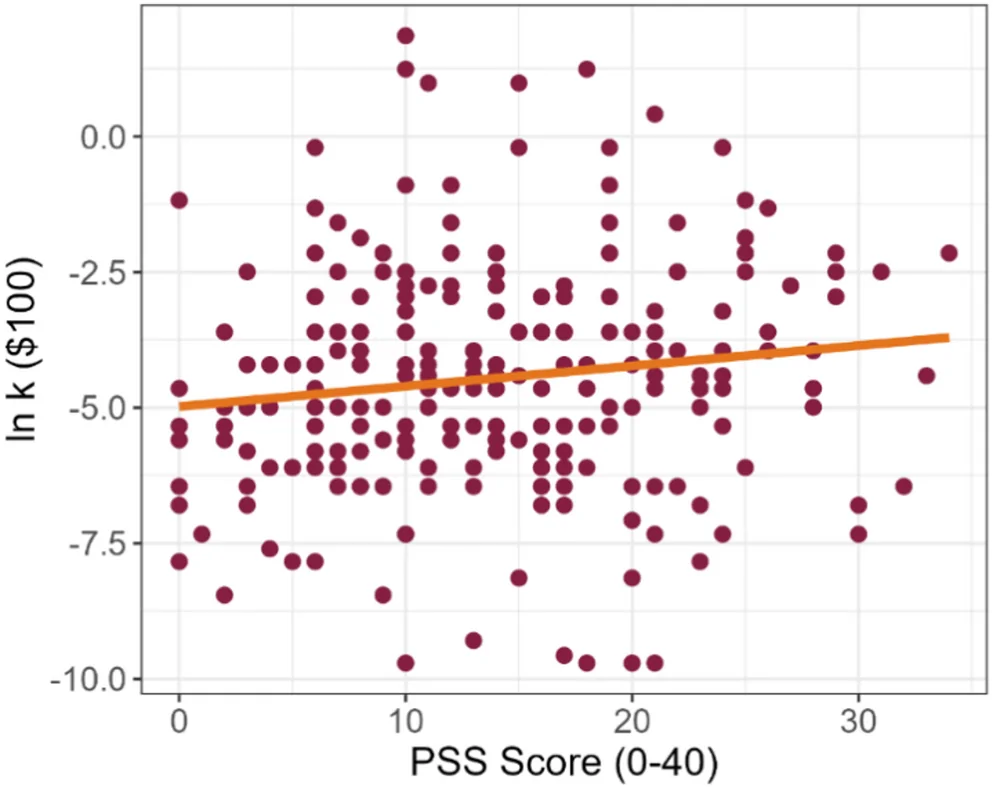
Scatter plot showing observed association between delay discounting (ln* k*, $100) and PSS scores

Significantly higher values of ln *k* were observed for the $100 magnitude (*M* = − 4.47, *SD* = 2.13) compared to the $10,000 magnitude (*M* = − 5.99, *SD* = 2.00), *t*(221) = 15.715, *p* < .001. Table 2 provides the results of linear regression analyses performed, using ln* k* ($100) as the outcome variable (N=222). In univariate regression, PSS score was found to be a significant predictor of ln* k* ($100) (standardized β = 0.14, *p* = .043), indicating that participants with higher levels of perceived stress had higher rates of DD in the $100 magnitude (see Fig. 1). Similar results were found for the multiple regression model, where there was a significant association between PSS score and ln* k* ($100) (standardized β = 0.16, *p* = .025). In the analysis of ln* k* ($10,000) (N=219), neither of the regression models included any significant predictors (see Supplemental Table 1). A sensitivity analysis for ln *k* ($10,000) was conducted with all participants (N=222), including the three previously identified outliers, with no significant results reported for either regression model (see Supplemental Table 2). There were no outliers in the $100 magnitude, so no sensitivity analysis was conducted for this magnitude.

**Table 2:** Univariate and multiple regression results, delay discounting (ln k, $100), N=222

	Univariate				Multiple		
Characteristic	Std. Beta	95% CI^1^	*p*-value	Std. Beta		95% CI^1^	*p*-value
Age	0.02	− 0.11, 0.16	0.725	0.04		− 0.09, 0.17	0.549
Education			0.120				0.198
2-year degree or less	–	–		–		–	
College grad/4− year degree	− 0.37	− 0.72, − 0.02		− 0.31		− 0.65, 0.03	0.072
Grad/professional degree	− 0.28	− 0.65, 0.08		− 0.20		− 0.55, 0.15	0.267
Gender			0.505				0.240
Male	–	–		–		–	
Female	− 0.09	− 0.36, 0.18		− 0.16		− 0.42, 0.11	
Household income ($1000)			0.325				0.335
100–149.9	–	–		–		–	
150–299.9	− 0.07	− 0.34, 0.21		0.00		− 0.28, 0.27	
300–449.9	− 0.23	− 1.00, 0.53		− 0.12		− 0.89, 0.65	
450+	− 1.04	− 2.19, 0.12		− 1.06		− 2.20, 0.09	
PSS score	0.14	0.00, 0.27	**0.043**	0.16		0.02, 0.30	**0.025**

^1^CI = Confidence Interval

Models have been standardized. Significant p values are bolded

#### Probability discounting

**Fig. 2:**
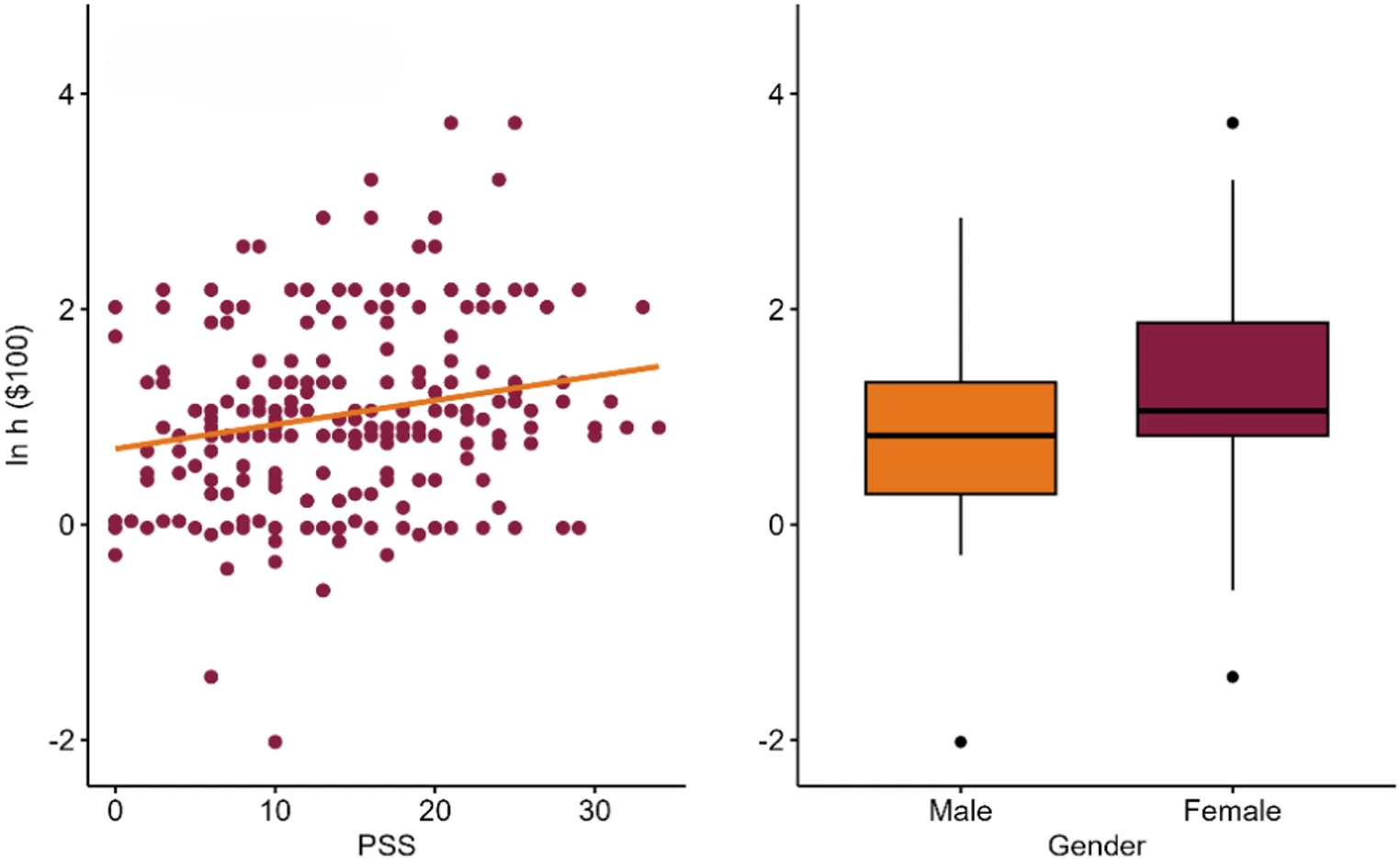
Scatter plot showing the association between probability discounting (ln* h*, $100) and PSS scores (left) and box plot showing the comparison of probability discounting (ln *h*, $100) in male and female participants (right)

Significantly lower values of ln *h* were observed for the $100 magnitude (*M* = 1.16, *SD* = 1.10) compared to the $10,000 magnitude (*M* = 1.69, *SD* = 1.30), *t*(221) = − 7.893, *p* < .001. Table 3 provides the results of univariate and multiple regression analyses performed, using ln* h* ($100) as the outcome variable (N=214). In univariate regression, PSS score was a significant predictor of ln *h* ($100) (standardized β = 0.19, *p* = .006), indicating that participants with higher PSS scores had higher rates of PD in the $100 magnitude. Gender (standardized β = 0.36, *p* = .008) was also found to be a significant predictor of ln *h* ($100), indicating that participants identifying as female had higher rates of PD in the $100 magnitude compared to participants identifying as male (see Fig. 2). In the multiple regression model, there was also a positive, significant association between ln *h* ($100) and PSS scores (standardized β = 0.16, *p* = .025). A sensitivity analysis for ln *h* ($100) was conducted with all participants (N=222), including the eight previously identified outliers, where the association with gender remained significant in both regression models (see Supplemental Table 3). In analysis of ln* h* ($10,000) (N=221), gender was significantly associated with ln *h* in univariate regression (standardized β = 0.31*, p* = .024), similar to the analysis for lower magnitude (see Supplemental Table 4), but there were no significant results reported for the multiple regression model. A sensitivity analysis for ln *h* ($10,000) was conducted with all participants (N=222), including the one previously identified outlier, where the significant association with gender persisted as well as a significant association with age in the multiple regression results (see Supplemental Table 5).

**Table 3:** Univariate and multiple regression results with outliers excluded, probability discounting (ln h, $100), N=214

	Univariate				Multiple		
Characteristic	Std. Beta	95% CI^1^	*p*-value	Std. Beta		95% CI^1^	*p*-value
Age	0.01	− 0.12, 0.15	0.857	0.05		− 0.09, 0.19	0.445
Education			0.135				0.351
2-year degree or less	–	–		–		–	
College grad/4-year degree	− 0.27	− 0.63, 0.10		− 0.21		− 0.57, 0.16	
Grad/professional degree	− 0.38	− 0.76, − 0.01		− 0.28		− 0.66, 0.10	
Gender			**0.008**				0.053
Male	–	–		–		–	
Female	0.36	0.09, 0.63		0.27		0.00, 0.54	
Household income ($1000)			0.573				0.596
100–149.9	–	–		–		–	
150–299.9	− 0.01	− 0.29, 0.27		0.01		− 0.27, 0.29	
300–449.9	− 0.03	− 0.85, 0.80		0.19		− 0.63, 1.01	
450+	− 0.83	− 1.98, 0.33		− 0.73		− 1.87, 0.40	
PSS score	0.19	0.05, 0.32	**0.006**	0.16		0.02, 0.30	**0.025**

^1^CI = Confidence Interval

Models have been standardized. Significant *p* values are bolded

## Discussion

The present study aimed to examine whether PSS scores, a common and well-validated measure of perceived stress, are associated with DD and PD in participants with higher income. Greater perceived stress was associated with DD and PD rates for $100, which is consistent with prior research showing that individuals with higher perceived stress levels show greater sensitivity to reward delay (Fields et al., 2009; Malesza, 2019) and uncertainty (Lempert et al., 2012). However, perceived stress was not associated with either DD or PD rates at the $10,000 reward magnitude, suggesting that the association between discounting and perceived stress is specific to smaller reward magnitudes. Finally, gender was significantly associated with PD ($100) in the univariate analysis and PD ($10,000) in both univariate and multiple regression analyses, where those identifying as female tended to favor smaller, guaranteed rewards over larger, uncertain rewards when compared with participants identifying as male.

By restricting the study sample to individuals with higher household income, these findings demonstrate that the association between perceived stress and both DD and PD is not strictly dependent on their mutual associations with socioeconomic disadvantage. Restriction of income as a potential confound is complementary to methods that control for potential confounding in the analysis phase (e.g., using ANCOVA or multiple regression), as each method has distinct advantages and disadvantages (Rothman et al., 2008). Specifically, sample restriction establishes control at the experimental design stage whereas the robustness of statistical control relies on accurate measurement and appropriate modeling of potential confounders, which are often limited when confounding is large or the sample size is insufficient to provide precise estimates. Conversely, statistical control combined with broad sampling enhances generalizability of findings compared to sample restriction. Thus, convergent evidence from both approaches strengthen conclusions.

Significant associations between discounting measures and perceived stress were restricted to the smaller of the two reward magnitudes. Although further research is required to understand these magnitude-selective effects, one possible explanation is related to the reward magnitude effect, in which we observed lower DD and higher PD rates for $10,000, consistent with prior findings (e.g., Cox et al., 2020; Green et al., 2013). This shift toward more extreme discounting rates at the $10,000 vs. $100 magnitude may have limited the tasks’ sensitivity to detect relatively smaller cross-sectional associations between perceived stress and individual differences in discounting and perceived stress.

In contrast, the associations between gender and PD remained significant at both reward magnitudes. It remains unclear why the associations between PD and gender, but not perceived stress, would be robust across $100 and $10,000. The persistence of gender in these results could be related to a number of factors, including previous findings showing gender differences in response to reward uncertainty where female participants have shown greater preference for smaller, more probable rewards compared to male participants (Cornwall et al., 2018). With DD, previous studies have not shown consistent results regarding gender differences, though there seems to be a tendency for males to discount future rewards at higher rates compared to females (see Lv et al., 2025 for meta-analysis). Future research should further explore these considerations and how they may be affected by stress, especially since the association between PD and perceived stress did not appear to be stable across our primary results and those of the sensitivity analysis.

The current study has several limitations. First, we specifically recruited non-healthcare workers in the present study, as this group was intended to serve as a comparison group in a separate line of research examining decision-making in primary care clinicians (e.g., Epling et al., 2026). Future studies should consider not using occupational restrictions, which may yield more generalizable conclusions. Second, while the Prolific research platform has yielded high-quality data, especially when compared with other popular crowdsourcing sites (Craft et al., 2022), there still remains bias in the sample by being collected entirely from a research volunteer platform. Third, the present study used brief adjusting procedures to assess DD and PD. Although the brief DD task has been validated against more commonly used measures (e.g., the adjusting-amount task; Koffarnus & Bickel, 2014) and shows good test–retest reliability (e.g., Gelino et al., 2024), there has been far less research on the validity of the brief PD task. Thus, the extent to which our present PD results may generalize when using other more commonly used assessments of PD requires further research. Nonetheless, the PD tasks used in the current study replicated the commonly reported magnitude effect in PD, providing some evidence of its validity. Lastly, the cross-sectional design of this survey-based study does not allow for any temporal relationship to be established between variables and causal relationships between discounting rates cannot be inferred.

For future studies, along with removing the income and employment restrictions from the current study, it would be beneficial to conduct a similar study through media other than crowdsourcing websites to determine whether these results could be replicated with other, more generalized groups. Regarding income restrictions, future studies should also seek to collect data regarding participants’ location and perceived financial distress, as cost of living and general expenses can vary greatly between different areas of the country and translate to varying levels of financial distress based on similar incomes. Additionally, it would be beneficial to see if the relationships observed from this sample persist when using other validated measures of stress, especially in various employment sectors. For example, multiple studies have observed higher stress levels in areas like healthcare, and it would be beneficial to explore concepts such as those attempted through measures of burnout and stress. Collecting data from participants over time is also a relevant goal for future research, in order to overcome the limitations of cross-sectional studies and determine whether these relationships are stable over time. Finally, an adjustment for multiple comparisons was not used in this analysis, which limits the opportunity for false negatives in this exploratory research on discounting and stress in an income-restricted sample. However, in order to obtain a full picture of the various types and magnitudes of discounting represented within the dataset, future confirmatory studies should control false positive rate consistent with the number of tests and specific hypotheses tested.

Overall, health-related decision-making is complex and involves the consideration of both short- and long-term consequences, as well as evaluations of risk, and understanding the potential relationships between these different types of discounting and perceived stress can inform future interventions to improve health outcomes across various clinical populations. Ultimately, we observed that in a higher-income sample, there are significant, positive relationships between discounting and perceived stress at the lower magnitudes for both delay and probability discounting.

## Electronic Supplementary Material

Below is the link to the electronic supplementary material.

Supplemental Table 1

Supplemental Table 2

Supplemental Table 3

Supplemental Table 4

Supplemental Table 5

## Data Availability

The dataset analyzed in this study has been made publicly available. This dataset can be found here: https://osf.io/j4mvy/overview?view_only=904bb268089049e0a8097849867f96b9 (accessed 19 January 2026).
