# Supplementary material for: Examining relationships between delay and probability discounting and perceived stress in a high-income online sample
Source: J Behav Med. 2026 Sep 3;49(6):1109–18. doi: 10.1007/s10865-026-00711-0 (PMC13748274; doi:10.1007/s10865-026-00711-0)
Supplement: Supplementary file 1 [file 10865_2026_711_MOESM1_ESM.docx]

| **Supplemental Table 1**  *Univariate and multiple regression results with outliers excluded, delay discounting (ln k, $10,000), N=219* | | | | | | |
| --- | --- | --- | --- | --- | --- | --- |
|  | Univariate | | | Multiple | | |
| Characteristic | Std. Beta | 95% CI*^1^* | *p*-value | Std. Beta | 95% CI*^1^* | *p*-value |
| **Age** | 0.02 | -0.12, 0.15 | 0.813 | 0.04 | -0.10, 0.18 | 0.619 |
| **Education** |  |  | 0.372 |  |  | 0.406 |
| *2-year degree or less* | — | — |  | — | — |  |
| *College grad/4-year degree* | -0.20 | -0.56, 0.15 |  | -0.17 | -0.54, 0.19 |  |
| *Grad/professional degree* | -0.02 | -0.39, 0.35 |  | 0.02 | -0.36, 0.40 |  |
| **Gender** |  |  | 0.238 |  |  | 0.123 |
| *Male* | — | — |  | — | — |  |
| *Female* | -0.16 | -0.43, 0.11 |  | -0.22 | -0.49, 0.06 |  |
| **Household income ($1000)** |  |  | 0.470 |  |  | 0.460 |
| *100-149.9* | — | — |  | — | — |  |
| *150-299.9* | -0.09 | -0.37, 0.19 |  | -0.04 | -0.32, 0.24 |  |
| *300-449.9* | -0.23 | -0.99, 0.54 |  | -0.14 | -0.92, 0.64 |  |
| *450+* | -0.84 | -2.00, 0.31 |  | -0.93 | -2.08, 0.23 |  |
| **PSS score** | 0.08 | -0.05, 0.21 | 0.230 | 0.12 | -0.03, 0.26 | 0.112 |
| *^1^*CI = Confidence Interval  *Note:* Models have been standardized. | | | | | | |
