# Supplementary material for: Examining relationships between delay and probability discounting and perceived stress in a high-income online sample
Source: J Behav Med. 2026 Sep 3;49(6):1109–18. doi: 10.1007/s10865-026-00711-0 (PMC13748274; doi:10.1007/s10865-026-00711-0)
Supplement: Supplementary file 2 [file 10865_2026_711_MOESM2_ESM.docx]

| **Supplemental Table 2**  *Results of sensitivity analysis with all participants included, delay discounting (ln k, $10,000), N=222* | | | | | | |
| --- | --- | --- | --- | --- | --- | --- |
|  | Univariate | | | Multiple | | |
| Characteristic | Std. Beta | 95% CI*^1^* | *p*-value | Std. Beta | 95% CI*^1^* | *p*-value |
| **Age** | 0.05 | -0.08, 0.19 | 0.432 | 0.08 | -0.06, 0.22 | 0.264 |
| **Education** |  |  | 0.589 |  |  | 0.642 |
| *2-year degree or less* | — | — |  | — | — |  |
| *College grad/4-year degree* | -0.11 | -0.46, 0.25 |  | -0.07 | -0.43, 0.29 |  |
| *Grad/professional degree* | 0.05 | -0.32, 0.42 |  | 0.08 | -0.30, 0.45 |  |
| **Gender** |  |  | 0.147 |  |  | 0.065 |
| *Male* | — | — |  | — | — |  |
| *Female* | -0.20 | -0.46, 0.07 |  | -0.26 | -0.53, 0.02 |  |
| **Household income ($1000)** |  |  | 0.496 |  |  | 0.451 |
| *100-149.9* | — | — |  | — | — |  |
| *150-299.9* | -0.08 | -0.35, 0.20 |  | -0.04 | -0.32, 0.24 |  |
| *300-449.9* | -0.26 | -1.02, 0.51 |  | -0.17 | -0.95, 0.60 |  |
| *450+* | -0.80 | -1.96, 0.35 |  | -0.92 | -2.08, 0.23 |  |
| **PSS score** | 0.09 | -0.05, 0.22 | 0.205 | 0.13 | -0.01, 0.28 | 0.064 |
| *^1^*CI = Confidence Interval  *Note:* Models have been standardized. | | | | | | |
