# Supplementary material for: Examining relationships between delay and probability discounting and perceived stress in a high-income online sample
Source: J Behav Med. 2026 Sep 3;49(6):1109–18. doi: 10.1007/s10865-026-00711-0 (PMC13748274; doi:10.1007/s10865-026-00711-0)
Supplement: Supplementary file 3 [file 10865_2026_711_MOESM3_ESM.docx]

| **Supplemental Table 3**  *Results of sensitivity analysis with all participants included, probability discounting (ln h, $100), N=222* | | | | | | | |
| --- | --- | --- | --- | --- | --- | --- | --- |
|  | Univariate | | | | Multiple | | |
| Characteristic | Std. Beta | 95% CI*^1^* | *p*-value | Std. Beta | | 95% CI*^1^* | *p*-value |
| **Age** | 0.08 | -0.06, 0.21 | 0.255 | 0.07 | | -0.06, 0.21 | 0.283 |
| **Education** |  |  | 0.089 |  | |  | 0.176 |
| *2-year degree or less* | — | — |  | — | | — |  |
| *College grad/4-year degree* | -0.35 | -0.71, 0.00 |  | -0.31 | | -0.67, 0.05 |  |
| *Grad/professional degree* | -0.38 | -0.75, -0.01 |  | -0.32 | | -0.69, 0.05 |  |
| **Gender** |  |  | **0.021** |  | |  | **0.048** |
| *Male* | — | — |  | — | | — |  |
| *Female* | 0.31 | 0.05, 0.57 |  | 0.27 | | 0.00, 0.55 |  |
| **Household income ($1000)** |  |  | 0.454 |  | |  | 0.522 |
| *100-149.9* | — | — |  | — | | — |  |
| *150-299.9* | 0.00 | -0.28, 0.27 |  | 0.03 | | -0.24, 0.31 |  |
| *300-449.9* | 0.37 | -0.40, 1.13 |  | 0.38 | | -0.39, 1.15 |  |
| *450+* | -0.75 | -1.91, 0.40 |  | -0.64 | | -1.79, 0.51 |  |
| **PSS score** | 0.03 | -0.10, 0.17 | 0.609 | 0.02 | | -0.12, 0.18 | 0.758 |
| *^1^*CI = Confidence Interval  *Note:* Models have been standardized. Significant *p* values are bolded. | | | | | | | |
