# Supplementary material for: Examining relationships between delay and probability discounting and perceived stress in a high-income online sample
Source: J Behav Med. 2026 Sep 3;49(6):1109–18. doi: 10.1007/s10865-026-00711-0 (PMC13748274; doi:10.1007/s10865-026-00711-0)
Supplement: Supplementary file 4 [file 10865_2026_711_MOESM4_ESM.docx]

| **Supplemental Table 4**  *Univariate and multiple regression results with outliers excluded, probability discounting (ln h, $10,000), N=221* | | | | | | | |
| --- | --- | --- | --- | --- | --- | --- | --- |
|  | Univariate | | | | Multiple | | |
| Characteristic | Std. Beta | 95% CI*^1^* | *p*-value | Std. Beta | | 95% CI*^1^* | *p*-value |
| **Age** | 0.12 | -0.02, 0.24 | 0.086 | 0.13 | | -0.01, 0.27 | 0.064 |
| **Education** |  |  | 0.178 |  | |  | 0.230 |
| *2-year degree or less* | — | — |  | — | | — |  |
| *College grad/4-year degree* | -0.21 | -0.57, 0.14 |  | -0.19 | | -0.55, 0.17 |  |
| *Grad/professional degree* | -0.35 | -0.71, 0.02 |  | -0.32 | | -0.69, 0.05 |  |
| **Gender** |  |  | **0.024** |  | |  | 0.056 |
| *Male* | — | — |  | — | | — |  |
| *Female* | 0.31 | 0.04, 0.57 |  | 0.26 | | -0.01, 0.53 |  |
| **Household income ($1000)** |  |  | 0.251 |  | |  | 0.237 |
| *100-149.9* | — | — |  | — | | — |  |
| *150-299.9* | 0.16 | -0.11, 0.44 |  | 0.20 | | -0.08, 0.47 |  |
| *300-449.9* | 0.53 | -0.23, 1.30 |  | 0.52 | | -0.25, 1.28 |  |
| *450+* | -0.57 | -1.72, 0.57 |  | -0.47 | | -1.61, 0.67 |  |
| **PSS score** | 0.01 | -0.12, 0.15 | 0.852 | 0.01 | | -0.13, 0.15 | 0.840 |
| *^1^*CI = Confidence Interval  *Note:* Models have been standardized. Significant *p* values are bolded. | | | | | | | |
