# Supplementary material for: Examining relationships between delay and probability discounting and perceived stress in a high-income online sample
Source: J Behav Med. 2026 Sep 3;49(6):1109–18. doi: 10.1007/s10865-026-00711-0 (PMC13748274; doi:10.1007/s10865-026-00711-0)
Supplement: Supplementary file 5 [file 10865_2026_711_MOESM5_ESM.docx]

| **Supplemental Table 5**  *Results of sensitivity analysis with all outliers included, probability discounting (ln h, $10,000), N=222* | | | | | | |
| --- | --- | --- | --- | --- | --- | --- |
|  | Univariate | | | Multiple | | |
| Characteristic | Std. Beta | 95% CI*^1^* | *p*-value | Std. Beta | 95% CI*^1^* | *p*-value |
| **Age** | 0.13 | 0.00, 0.26 | 0.059 | 0.14 | 0.00, 0.27 | **0.045** |
| **Education** |  |  | 0.191 |  |  | 0.248 |
| *2-year degree or less* | — | — |  | — | — |  |
| *College grad/4-year degree* | -0.24 | -0.60, 0.11 |  | -0.22 | -0.57, 0.14 |  |
| *Grad/professional degree* | -0.34 | -0.71, 0.03 |  | -0.31 | -0.68, 0.06 |  |
| **Gender** |  |  | **0.014** |  |  | **0.039** |
| *Male* | — | — |  | — | — |  |
| *Female* | 0.33 | 0.07, 0.59 |  | 0.28 | 0.02, 0.55 |  |
| **Household income ($1000)** |  |  | 0.219 |  |  | 0.185 |
| *150 or less* | — | — |  | — | — |  |
| *150-299.9* | 0.19 | -0.09, 0.46 |  | 0.22 | -0.05, 0.50 |  |
| *300-449.9* | 0.55 | -0.22, 1.31 |  | 0.54 | -0.22, 1.30 |  |
| *450+* | -0.53 | -1.68, 0.62 |  | -0.43 | -1.57, 0.70 |  |
| **PSS score** | 0.02 | -0.11, 0.15 | 0.760 | 0.02 | -0.12, 0.16 | 0.740 |
| *^1^*CI = Confidence Interval  *Note:* Models have been standardized. Significant *p* values are bolded. | | | | | | |
